# The Effect of Education on the Assessment of Optic Nerve Head Photographs for the Glaucoma Diagnosis

**DOI:** 10.1186/1471-2415-11-12

**Published:** 2011-05-19

**Authors:** Sabina Andersson, Anders Heijl, Andreas G Boehm, Boel Bengtsson

**Affiliations:** 1 Department of Clinical Sciences, Ophthalmology, Malmö, Lund University, Sweden; 2 Department of Ophthalmology, Elblandklinikum Radebeul, Radebeul, Germany; 3 Department of Ophthalmology, University of Dresden, Dresden, Germany

## Abstract

**Background:**

To evaluate the effect of one lesson of continuing medical education (CME) of subjective assessment of optic nerve head appearance on sensitivity and specificity for the diagnosis of glaucoma.

**Methods:**

Ophthalmologists and residents in ophthalmology attending an international glaucoma meeting arranged at Malmö University Hospital, Malmö, Sweden, were asked to grade optic nerve head (ONH) photographs of healthy and glaucomatous subjects at two sessions separated by a lecture on glaucoma diagnosis by ONH assessment. Each grader had access to an individual portfolio of 50 ONH photographs randomly selected from a web-based data bank including ONH photographs of 73 glaucoma patients and 123 healthy subjects. The individual portfolio of photographs was graded before and after the lecture, but in different randomized order.

**Results:**

Ninety-six doctors, 91% of all attending the meeting, completed both assessment sessions. The number of correct classifications increased from 69 to 72% on the average. Diagnostic sensitivity increased significantly (p < 0.0001) from 70% to 80%, and the number of photographs classified as uncertain decreased significantly (p < 0.0001) from 22% to 13%. Specificity remained at 68%, and intra-grader agreement decreased.

**Conclusion:**

CME had only a small effect on the assessment of ONH for the glaucoma diagnosis. Sensitivity increased and the amount of uncertain classifications decreased, while specificity was unchanged.

## Background

In clinical practice assessment of glaucomatous changes of the optic nerve head (ONH) may be the first step to detect glaucoma. Inspection of the posterior pole by e.g. ophthalmoscopy is a routine examination in most eye clinics. A suspect ONH appearance often directly leads to further examinations including perimetry and tonometry, or to referral of the patient. ONH assessment is sometimes a difficult task, particularly at early stages of glaucoma, requiring careful observation and knowledge about variability of optic disc appearance among healthy subjects and the characteristics of glaucomatous damage. Large and pale excavations at advanced stages of glaucoma have been recognized since the ophthalmoscope was introduced in the 1850's. Cup to disc ratios [1] was an attempt to quantify the excavation of ONH, and a number of signs typical for glaucoma have been described, e.g., saucerization of the disc [2], thinning of the neural rim [3], focal notching [4]. Disc hemorrhages, first described by Bjerrum in 1889 [5] are now considered a relatively hard sign of glaucoma. The ISNT rule suggested by Jonas and co-workers 1988 [6] compares the width of the neural rim in the interior, superior, nasal and temporal parts of the ONH. The size of the ONH is important for detection of glaucoma [7,8]. Glaucomatous eyes having small ONHs are more likely to be classified as normal than glaucomatous eyes with normal or large ONHs. On the contrary, healthy eyes with large OHNs are more likely to be classified as glaucomatous than healthy eyes with normal or small ONHs [9,10].

During the last two decades new computerized image techniques measuring and analyzing ONHs were developed. However, these image techniques are typically applied on patients already having a diagnosis of glaucoma or suspect glaucoma, and are not standard examinations in patients visiting eye departments/clinics for reasons other than suspect glaucoma or glaucoma. Therefore, subjective evaluation of the ONH, by e.g. ophthalmoscopy, is still important and often remains the first step when diagnosing previously undetected glaucoma.

A number of earlier studies have reported diagnostic accuracy of subjective assessment of the ONH [11-17], but to our knowledge no studies have reported on the effect of continuing medical education (CME) on diagnostic accuracy of subjective optic disc assessment of residents in ophthalmology or of ophthalmologists.

The aim of our study was to evaluate the effect of one hour of CME of ophthalmologists on the subjective grading of ONH photographs from glaucoma patients and healthy individuals.

## Methods

In conjunction with an international glaucoma meeting at Malmö University Hospital, Malmö, Sweden in March 2008, attending doctors were asked to grade ONH photographs using a web-based protocol at two different sessions. The two sessions were separated b y a lecture in glaucoma diagnosis by ONH assessment. Participation was voluntary and the first assessment was made before the start of the meeting, while the second assessment was performed after the lecture. No time limits for the assessments were given. The participants were each asked to classify 56 ONH photographs into one of three different categories: glaucomatous, normal or uncertain. Fifty photographs were unique and 6 duplicates. They were also asked to report whether they considered themselves to be a glaucoma expert, general ophthalmologist with special interest in glaucoma, general ophthalmologist, or ophthalmologist with another subspecialty. The participants were also asked to report their experience in glaucoma by choosing one of the following alternatives; lack of experience, less experienced, experienced or very experienced, and to report the average number of glaucoma patients seen per month.

The research project followed the tenets of the Declaration of Helsinki and was approved by the regional ethical review board in Lund, Sweden, vetting the ethics of research involving humans.

### Photographs - glaucoma patients and healthy subjects

ONH photographs from glaucoma patients and healthy subjects were retrieved from an existing database. The database has been described previously [18,19]. All glaucoma subjects were patients at the Department of Ophthalmology, Malmö University Hospital. All had a diagnosis of glaucoma with reproducible visual field defects and the Glaucoma Hemifield Test outside normal limits on standard automated perimetry using the 30-2 Full Threshold program of the Humphrey Field Analyzer (Carl Zeiss Meditec, Dublin, Calif, USA). To be included we required that the interval between the visual field test and photograph should be less than 12 months, and that the image quality was good enough to enable a fair evaluation. Photographs with obvious artifacts, e.g., the shutter half way down or prominent reflections, were excluded. One eye per patient, the one with the best perimetric Mean Deviation (MD) value, was selected. A total of 73 photographs from 73 glaucoma patients were included. Mean age for these patients was 70 years, ranging from 50 to 87 and the mean MD was -7.39 dB, ranging from -19.56 to +1.01 dB.

The healthy subjects were randomly selected individuals living in the city of Malmö, Sweden [18]. At the time for the data collection all supposedly healthy subjects underwent a thorough ophthalmic examination including determination of refraction and visual acuity, Goldmann applanation tonometry, slitlamp examination and recording of ophthalmic and general medical history. Inclusion criteria were corrected visual acuity ≥ 0.8, intraocular pressure <22 mmHg. Exclusion criteria were history of serious eye trauma or surgery, previous or current serious eye disease or any neurological disease. Again we excluded ONH photographs with poor quality or with obvious artifacts. To match the age of the glaucoma patients all subjects younger than 50 years of age at the time for the data collection were excluded. One eye per subject was randomly chosen and a total of 128 normal eyes were included. The mean age of the healthy subjects was 66 years, ranging from 51 to 79.

All photographs were obtained using the same fundus camera, a Carl Zeiss (Model 60 306, Oberkochen, West Germany) with standard settings (aperture 5.5, flash strength 120-240 Ws), using Kodachrome 64 slide film. The photographs were digitized using Nikon Super Coolscan 4000 ED diapositive scanner with the highest resolution of 4000 Dots Per Inch (dpi). The size was thereafter changed to 1400 × 1024 pixels with a resolution of 72 dpi to create a database that can be accessed through a web interface. This web-based database included the 201 ONH photographs. When logging in to the database a random subset of 50 photographs was automatically selected, and a unique mix of photographs was created for each grader. To be able to test intra-grader agreement, 6 ONH photographs randomly selected in each subset of 50 photographs were duplicated, thus creating an individual mix of 56 ONH photographs. Each grader then classified the same 56 photographs before and after the lecture, but the photographs were sorted in different randomized orders. The median number of photographs from glaucoma subjects was 19 (ranging from 12 to 24), and 31 from healthy individuals (ranging from 26 to 38).

### The lecture

The lecture in ONH assessment for glaucoma diagnosis was performed by one of the authors (AGB). It was a one hour lecture based on Dr. Remo Susanna's course "How to assess the optic nerve head and the retinal nerve fiber layer in glaucoma", with definition of five rules to detect glaucoma [20]. The lecture presented a systematic approach to evaluate optic discs with regards to glaucoma detection and focused particularly on the evaluation of optic disc size, neuroretinal rim, retinal nerve fiber layer, parapapillary atrophy and disc hemorrhages. All ONH photographs shown in the lecture were selected by the lecturer and were extracted from an independent database of glaucoma patients and healthy subjects collected at the Department of Ophthalmology, University of Dresden, Dresden, Germany. The lecture was ended with a short training session to use this systematic approach on 15 ONH photographs; nine glaucomatous ONHs, four healthy ONHs, one ONH with drusen and one ONH with optic pit.

### Analyses

Grader identity was masked in all analyses. Results were calculated for those graders performing assessments both prior to and after the lecture. Diagnostic accuracy was calculated in total as the percentage of correct classification among the 50 ONH photographs of each grader, and as sensitivity and specificity. The "uncertain" classifications were not included in the calculation of diagnostic accuracy. The change between sessions in diagnostic accuracy and number of uncertain classification for the whole group of graders was analyzed by one-sample t-test of differences. Mean values were calculated and reported for each subgroup, however, no comparisons for statistical differences between subgroups were performed since the number of participants was very low in some groups. The intra-grader variability was calculated by kappa statistics and interpreted by the rules suggested by Altman [21]:

No agreement - less than 0

Poor agreement - 0 than 0.20

Fair agreement - 0.20 to 0.40

Moderate agreement - 0.40 to 0.60

Good agreement - 0.60 to 0.80

Very good agreement - 0.80 to 1.00

Change in kappa values was tested by one sample t-test.

## Results

One hundred and forty-one doctors attended the meeting, and 105 participated in the first ONH assessment prior to the lecture in ONH assessment. Ninety-six doctors completed both assessment sessions: thus our results are based on those 96 physicians. The largest group, 39% of all participants, consisted of ophthalmologists with special interest in glaucoma, the second largest, 29%, was general ophthalmologists, 16% reported themselves to be glaucoma specialist, 3% other subspecialty and 7% were residents. The remaining 6% percent of the 96 participants did not report any group affiliation. The majority, 80%, of the participating doctors stated themselves to be very experienced or experienced in glaucoma care, and 77% had been active as clinicians for at least 10 years, Table 1.

**Table 1:** Self reported clinical experience

Number of glaucoma patients seen per month		Experience of glaucoma		Years as clinician	
≤10	10%	Lack experience	1%	0-4	8%
11-50	44%	Less experience	19%	5-9	15%
>50	46%	Experienced	65%	10-14	24%
		Very experienced	15%	>15	53%

**Figure 1:**
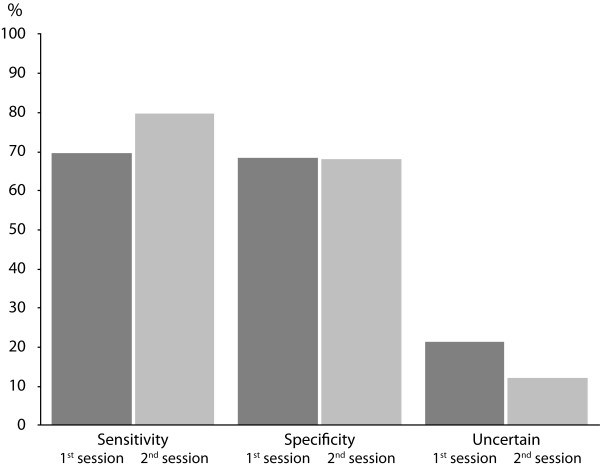
**Sensitivity and specificity for all graders before and after the lecture in ONH assessment**. The mean sensitivity of all 96 graders increased with 10% (p < 0.0001) from first to the second session. Specificity remained at the same level and the number of uncertain classifications decreased with 9% (p < 0.0001) from 22% before the lecture, to 13.0% after.

After the lecture the number of correct classifications improved significantly (p = 0.001) from 68 to 72%. The improvement in sensitivity was notable, from 70 to 80%, (0 <0.0001), while specificity remained at the same level at 68%. The number of uncertain classifications decreased significantly (p < 0.0001) from 22 to 13% after the lecture. Figure 1. Eleven percent of the photographs of healthy subjects initially classified as uncertain were correctly classified as normal at the second assessment. A similar number of photographs of glaucomatous eyes, 12%, initially classified as uncertain, were correctly classified as glaucomatous at the second assessment. The number of normal eyes initially erroneously classified as glaucomatous and correctly classified as normal at the second assessment (3%), was similar to the number of glaucomatous eyes initially erroneously classified as normal but correctly classified as glaucoma at the second assessment (4%). The difference between normal and glaucomatous eyes was that more initially correctly classified normal eyes were erroneously classified as glaucoma or uncertain in the second assessment (15%), than were initially correctly classified glaucomatous eyes, which were erroneously classified as normal or uncertain at the second assessment, 4%.

Among the groups sensitivity increased most, 26%, in the group of three doctors having other subspeciality, and in least, 8%, in the group with glaucoma experts, Table 2. Specificity was almost the same in the first and second assessment session in all subgroups, except for residents, where the specificity decreased with 9%, Table 2. The number of photographs classified as uncertain decreased in all subgroups. The smallest change was in the group of residents from 17% to 10%, Table 2.

**Table 2:** Average sensitivity, specificity and number of uncertain classifications divided by reported affiliation/subgroup

		Sensitivity		Specificity		Uncertain	
		**1**^**st **^ **session**	**2**^**nd **^ **session**	**1**^**st **^ **session**	**2**^**nd **^ **session**	**1**^**st **^ **session**	**2**^**nd **^ **session**

**All**	n = 96	70%	80%	68%	68%	22%	13%

**Glaucoma expert**	n = 15	77%	85%	67%	68%	17%	6%

**General ophthalmologist** **with special interest in** **glaucoma**	n = 37	72%	81%	72%	72%	20%	12%

**General ophthalmologist**	n = 28	64%	74%	67%	67%	26%	16%

**Other subspecialist**	n = 3	55%	81%	71%	67%	31%	15%

**Resident**	n = 7	72%	84%	60%	51%	17%	10%

**Not specified**	n = 6	70%	81%	70%	74%	22%	12%

Intra-grader agreement, expressed as kappa, was on the average moderate, both before (0.53) and after (0.43) the lecture. The change was significant, p = 0.02. In the first session 17 graders showed very good agreement, while three graders showed no agreement. In the second session only five graders showed very good agreement and seven graders no agreement.

## Discussion

The effect of re-training of ONH assessment was small, but positive. Sensitivity improved and the number of uncertain classifications decreased, while specificity remained at the same level. The number of physicians in the different subgroups was too small for any statistical analysis, but as expected sensitivity was highest, both before and after the training (77% and 87% respectively) in the group of glaucoma experts. It may be more surprising that the effect of CME on sensitivity was of similar magnitude, approximately 10% improvement, in the expert group as in the other subgroups (Table 2) except for those with other subspecialty where sensitivity increased from 55% to 81%, but this group included 3 ophthalmologists only.

Uncertain classifications in the group with all graders, decreased significantly in number after the training session. A decrease was seen for all the subgroups, and somewhat surprisingly, even the glaucoma experts showed a marked decrease in the amount of uncertain classifications. The move from the uncertain alternative in the first session was almost equally divided to the glaucoma and healthy alternatives in the second session. This improved both sensitivity and specificity, but then the move of correctly classified healthy ONHs in first session to erroneously classifications in the second session was about four times larger than the move of correctly classified glaucomatous ONHs in the first session to erroneously classifications in the second session, which resulted in improved sensitivity and no change in specificity on the average.

Few articles have described effects of CME on ONH assessment for the diagnosis of glaucoma. To our knowledge the current study is the first to investigate the effect of CME for the glaucoma diagnosis in ophthalmologists with different experience. This kind of CME is often offered at ophthalmic meetings, with the aim of improving diagnostic performance of the auditorium. Margolis and co-workers reported in 1989 [22] effects of an educational program in ONH assessment for residents in internal medicine and practicing internists. In this study both sensitivity and specificity improved significantly with 10 to 20% in the two groups. In the current study sensitivity, but not specificity, improved after the lecture. One reason may be that the short training session at the end of the lecture only included four photographs of healthy ONHs. Perhaps an extended education on normal variability of ONH appearance would have improved the specificity, similar to that seen on sensitivity. Our ONH photographs came from glaucoma patients with different disease severities from very early glaucoma to those with severe glaucomatous damage. The collection of ONH photographs of the healthy subjects was performed in a population-based sample including a wide range of normal ONH appearances, and not only obviously healthy looking ONHs.

Sheen et al. [23] also reported positive effects of education on disc assessment, but this time performed by medical students. In this study the results parameter was inter-grader agreement, calculated as the standard deviation of cup/disc ratios differences, between students and an expert observer. In the current study we have no result parameter to compare with this inter-grader agreement outcome, but we measured intra-grader agreement before and after the education lecture. Our result was negative, since the intra-grader agreement decreased.

The use of digitized photographs in our study enabled the graders to interpret images without any stress caused by time limits and also strongly facilitated the randomization of images to each grader. Digitized photographs have been shown to be a reasonable alternative and comparable with traditional slide photographs. Stone et al. recently reported that primary digital or scanned optic disc images were suitable substitutes for traditional slide photographs [24]. Within retinal diseases such as diabetic retinopathy and macular degeneration there are several image quality studies comparing digital and digitized images with slide photographs, concluding that there are a close agreement [25-28].

A positive learning effect of teaching could be anticipated, and of course the result depends both on the teacher, the auditorium and the format for the training. In our study the total improvement after the training lesson was significant, however the change was a marginal 4% only. The lecture format only may not be the optimal way to train ophthalmologist to read ONH for the diagnosis of glaucoma. A two-way communication, e.g. interactive education, instead of a one-way lecture may possibly yield a somewhat better result.

## Conclusions

Training produced small positive changes in diagnostic performance, also among glaucoma experts. Sensitivity increased significantly, while specificity was unchanged, and the number of uncertain classifications decreased significantly.

## Competing interests

The authors declare that they have no competing interests.

## Authors' contributions

The practical arrangements of the project and its implementation was carried out by SA with aid from AH and BB. SA performed the calculations of data and the statistical analyses in collaboration with BB. The manuscript was drafted by SA but AH and BB, who designed the study, also contributed substantially with the work of the manuscript. The lecture in ONH assessment was planned and carried out by AGB who also drafted the lecture session of the manuscript. All authors read and approved the final manuscript.

## Pre-publication history

The pre-publication history for this paper can be accessed here:

http://www.biomedcentral.com/1471-2415/11/12/prepub
